# Chicken cecal microbiota reduces abdominal fat deposition by regulating fat metabolism

**DOI:** 10.1038/s41522-023-00390-8

**Published:** 2023-05-30

**Authors:** Yan Chen, Muhammad Akhtar, Ziyu Ma, Tingwei Hu, Qiyao Liu, Hong Pan, Xiaolong Zhang, Abdallah A. Nafady, Abdur Rahman Ansari, El-Sayed M. Abdel-Kafy, Deshi Shi, Huazhen Liu

**Affiliations:** 1 Key Laboratory of Agricultural Animal Genetics, Breeding and Reproduction of Ministry of Education, Huazhong Agricultural University, Wuhan, 430070 P.R. China; 2 Section of Anatomy and Histology, Department of Basic Sciences, College of Veterinary and Animal Sciences (CVAS) Jhang, University of Veterinary and Animal Sciences (UVAS), Lahore, Pakistan; 3 Animal Production Research Institute (APRI), Agricultural Research Center (ARC), Ministry of Agriculture, Giza, Egypt; 4 Department of Preventive Veterinary Medicine, College of Animal Science and Veterinary Medicine, Huazhong Agricultural University, Wuhan, 430070 P.R. China

**Keywords:** Microbiome, Microbiota

## Abstract

Cecal microbiota plays an essential role in chicken health. However, its contribution to fat metabolism, particularly in abdominal fat deposition, which is a severe problem in the poultry industry, is still unclear. Here, chickens at 1, 4, and 12 months of age with significantly (*p* < 0.05) higher and lower abdominal fat deposition were selected to elucidate fat metabolism. A significantly (*p* < 0.05) higher mRNA expression of fat anabolism genes (*ACSL1*, *FADS1*, *CYP2C45, ACC*, and *FAS*), a significantly (*p* < 0.05) lower mRNA expression of fat catabolism genes (*CPT-1* and *PPARα*) and fat transport gene *APOAI* in liver/abdominal fat of high abdominal fat deposition chickens indicated that an unbalanced fat metabolism leads to excessive abdominal fat deposition. *Parabacteroides*, *Parasutterella*, *Oscillibacter*, and *Anaerofustis* were found significantly (*p* < 0.05) higher in high abdominal fat deposition chickens, while *Sphaerochaeta* was higher in low abdominal fat deposition chickens. Further, Spearman correlation analysis indicated that the relative abundance of cecal *Parabacteroides*, *Parasutterella*, *Oscillibacter*, and *Anaerofustis* was positively correlated with abdominal fat deposition, yet cecal *Sphaerochaeta* was negatively correlated with fat deposition. Interestingly, transferring fecal microbiota from adult chickens with low abdominal fat deposition into one-day-old chicks significantly (*p* < 0.05) decreased *Parabacteroides* and fat anabolism genes, while markedly increased *Sphaerochaeta* (*p* < 0.05) and fat catabolism genes (*p* < 0.05). Our findings might help to assess the potential mechanism of cecal microbiota regulating fat deposition in chicken production.

## Introduction

In the poultry industry, the artificial selection of chickens for commercial purposes through genetic breeding technology and a higher energy diet unprecedentedly enhanced the growth rate and feed conversion of broilers^1^. However, rapidly growing broilers are often accompanied by excessive abdominal fat deposition^2^, which is an unfavorable trait both for consumers and producers, and more than 85% of abdominal fat is useless for the body because it is considered the wastage of dietary energy^3^. A recent report indicated that broilers produced ≈3 million tons of abdominal fat around the world annually, which results in >$2.7 billion economic loss in the poultry industry^4^, leading to a key hindrance to profitable farming^5^. Although it is an appreciable energetic component, it has to be removed during evisceration and is considered a waste in chicken meat production^6^. Abdominal fat deposition decreases feed utilization, reduces the reproduction performance of laying hens, negatively affects the slaughtering process, and causes environmental pollution^2,7,8^. It also increases fat contents in chicken meat, which increases the risk of human cardiovascular diseases^9^. Researchers have found that biologically, the abdominal adipocytes are more active cells exhibiting a higher (0.82) heritability rate than bodyweight, breast, and leg muscles^5^, resulting in fat accumulation. It is also reported that abdominal fat weight and body weight had a strong positive correlation, which is hindering genetic selection against fatness traits in chickens^4^. The excessive fat deposition has become a puzzle and also an emerging concern in the recent decades. Therefore, understanding the mechanism which leads to excessive fat deposition has become an important question.

The host gut harbors ~80% of the symbiont microorganisms, of which 99% are bacteria, called gut microbiota^10–13^. It has been established that gut microbiota could play a significant regulatory role in fat deposition and obesity^4,14^. Evidence revealed that colonization of the obese microbiota promoted fat deposition in mice^15^. For example, a higher abundance of *Methanobrevibacter* and *Faecalibacterium*, while a lower abundance of *Akkermansia* increases fat deposition^4,6^. Further studies indicated that gut microbiota influences and modulates fat metabolism, and importantly contributes to nutrient utilization, generating additional harvestable energy and resulting in abdominal fat deposition^6,16^. For instance, *Enterococcus faecium* increases fatty acid synthase (*FAS*) and acetyl-CoA carboxylase (*ACC*) secretion in chicken liver^17^, and elevated *FAS* and ACC increase fatty acid production, which incorporates into triglyceride and increases fat deposition^18^. *Klebsiella* and *Escherichia*-*Shigella* possess lipogenesis characteristics, and their higher abundance increases total cholesterol, low-density lipoprotein, and triglyceride concentrations in serum, which facilitate fat accumulation^19^. On the other hand, some microbiota such as *Mucispirillum schaedleri* decreases fat deposition in chickens^4^, and *Sphaerochaeta* is found enriched in lean chickens^14^. *Lactobacillus johnsonii* BS15 decreases fat deposition through lipoprotein lipase (*LPL*) activity and improves fat catabolism in broilers^20^. Abundant *Microbacterium* and *Sphingomonas* in chicken were positively related to fat catabolism genes in muscles and liver, which potentially reduce fat storage^21^. Previous studies indicated that gut microbiota not only can increase fat deposition but also can decrease fat deposition^4,14^. In the complex network of gut microbial communities, dynamically the highest bacterial diversity is observed in the cecum^22^. Therefore, what is cecal bacterial composition and what kind of cecal bacteria could reduce abdominal fat deposition, and how they regulate fat metabolism has become an interesting question.

To address this concern, chickens (Turpan cockfighting × White Leghorn) at three different ages (1 month, 4 months, and 12 months) with significantly different abdominal fat deposition were used in the present study. The fat metabolism levels, cecal microbial communities, and the abundances of different bacteria were compared between high and low abdominal fat deposition chickens. Spearman correlation analysis was used to find the relationship between cecal microbiota and abdominal fat deposition. Furthermore, transferring fecal microbiota from adult healthy chickens with low abdominal fat deposition into 1-day-old white feather broiler chicks was performed to verify whether gut microbiota could regulate chicken fat deposition, and the fat metabolism levels in the liver and abdominal adipose tissues were also compared.

## Results

### The abdominal fat deposition is significantly different between high and low abdominal fat deposition chickens

**Fig. 1:**
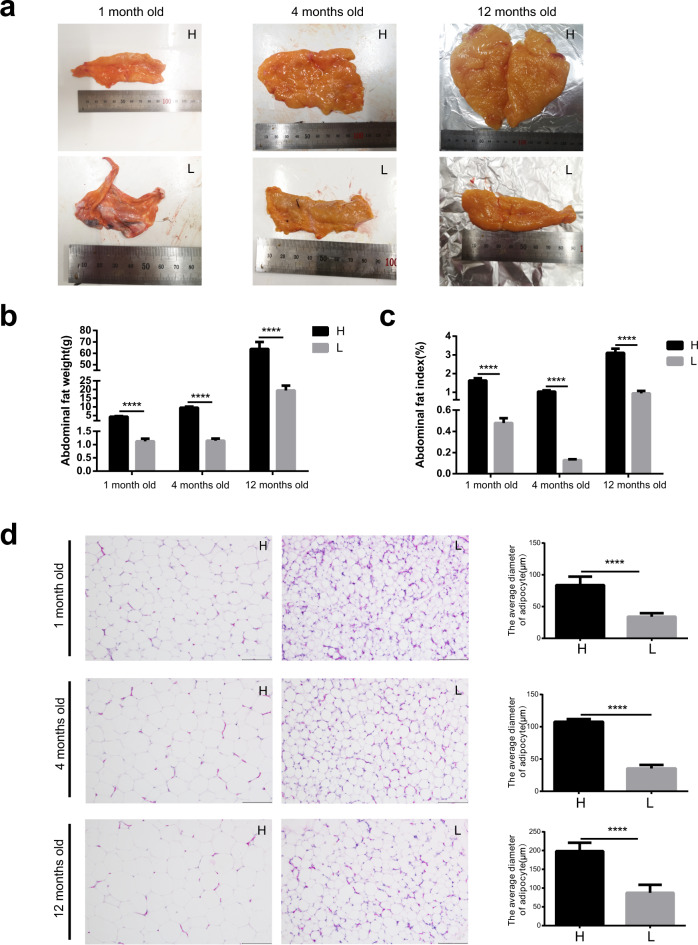
Analysis of the differences of abdominal fat deposition between high and low abdominal fat deposition chickens at different months. **a** The comparison of abdominal fat volume between high and low abdominal fat deposition chickens at different months. **b** The comparison of abdominal fat weight between high and low abdominal fat deposition chickens at different months. **c** The comparison of abdominal fat index between high and low abdominal fat deposition chickens at different months. **d** HE staining sections of fat abdominal adipose tissues and the comparison of an average diameter of adipocytes in high and low abdominal fat deposition chickens at different months. Scale bars = 100 μm. H represents high abdominal fat chickens (*n* = 10), and L represents low abdominal fat chickens (*n* = 10). Statistical significance between groups was determined by unpaired Student’s *t* tests. All data were presented as mean ± SEM. ****p < 0.0001.

Based on the abdominal fat index, the chickens (Turpan cockfighting × White Leghorn) at different ages (1 month old, 4 months old, and 12 months old) were divided into high abdominal fat deposition chickens (H group) and low abdominal fat deposition chickens (L group) respectively. The abdominal fat volume (Fig. 1a), abdominal fat weight (H vs L, 1 month old: 4.33 ± 0.31 g vs 1.12 ± 0.09 g; 4 months old: 9.58 ± 0.56 g vs 1.15 ± 0.08 g; 12 months old: 63.77 ± 6.19 g vs 19.46 ± 2.77 g) (unpaired Student’s *t* tests, *p* < 0.0001) (Fig. 1b), and abdominal fat index (H vs L, 1 month old: 1.63 ± 0.12% vs 0.48 ± 0.45%; 4 months old: 1.04 ± 0.07% vs 0.13 ± 0.01%; 12 months old: 3.11 ± 0.22% vs 0.94 ± 0.13%) (unpaired Student’s *t* tests, *p* < 0.0001) (Fig. 1c) were significantly higher in high abdominal fat deposition chickens. Hematoxylin and eosin (HE) staining results showed that the average diameter of abdominal adipocytes was significantly higher in high abdominal fat deposition chickens than that in low abdominal fat deposition chickens (unpaired Student’s *t* tests, *p* < 0.0001) (Fig. 1d). The above results showed that there were significant differences in fat deposition between high and low abdominal fat deposition chickens.

### The fat metabolism is significantly different between high and low abdominal fat deposition chickens

**Fig. 2:**
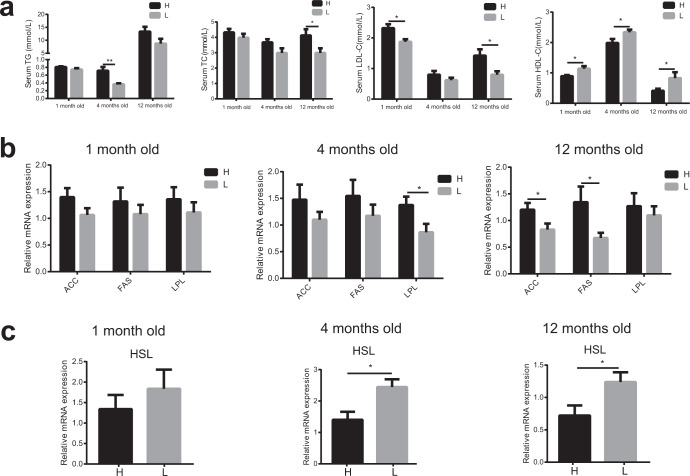
Analysis of fat metabolism differences in blood and abdominal fat between high and low abdominal fat deposition chickens at different months. **a** The comparison of serum triglycerides (TG) concentrations (mmol/L), serum total cholesterol (TC) concentrations (mmol/L), serum LDL-C concentrations (mmol/L), and serum HDL-C concentrations (mmol/L) between high and low abdominal fat deposition chickens at 1, 4, and 12 months. **b** The comparison of relative mRNA expression of fat synthesis related genes between high and low abdominal fat deposition chickens at 1, 4, and 12 months (q-PCR). **c** The comparison of relative mRNA expression of fat catabolism related genes between high and low abdominal fat deposition chickens at 1, 4, and 12 months (q-PCR). H represents high abdominal fat chickens (*n* = 10), and L represents low abdominal fat chickens (*n* = 10). Statistical significance between groups was determined by unpaired Student’s *t* tests. All data were presented as mean ± SEM. *p < 0.05, **p < 0.01.

**Fig. 3:**
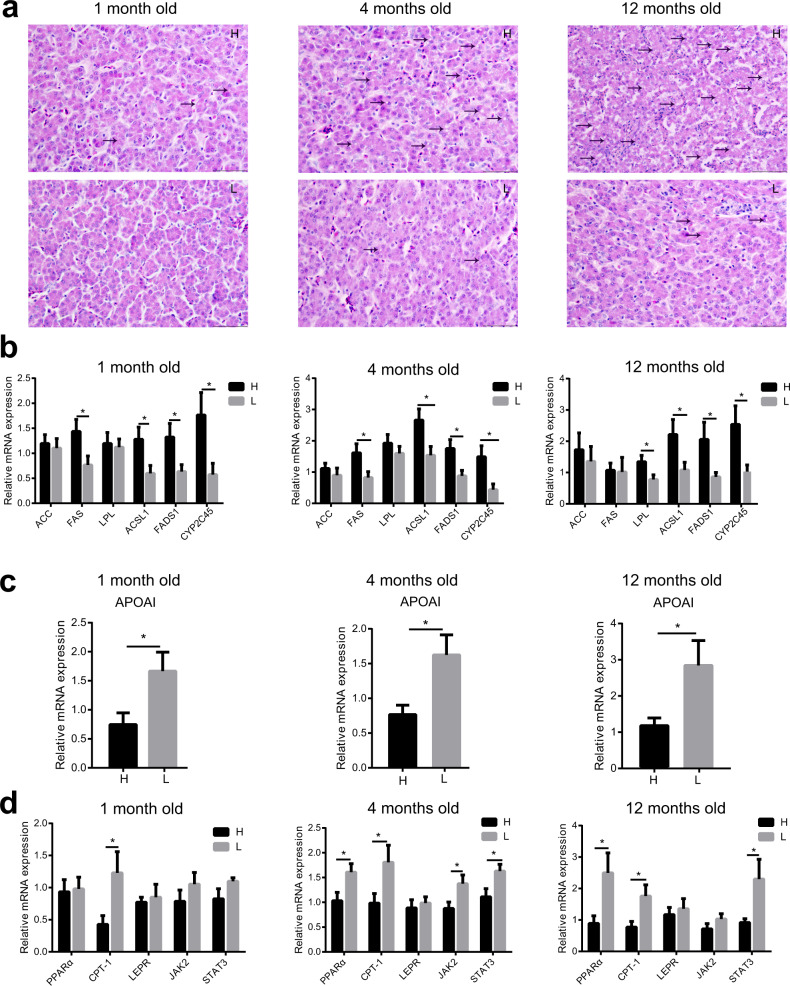
Analysis of fat metabolism differences in liver between high and low abdominal fat deposition chickens at different months. **a** HE staining sections of fat content in hepatocytes of the chickens at 1, 4, and 12 months. The fat droplets (white) are indicated with the arrows in the figures. **b** The comparison of relative mRNA expression of fat synthesis related genes between the high and low abdominal fat deposition chickens at 1, 4, and 12 months (q-PCR). **c** The comparison of relative mRNA expression of fat transport related genes between the high and low abdominal fat deposition chickens at 1, 4, and 12 months (q-PCR). **d** The comparison of relative mRNA expression of fat catabolism related genes between the high and low abdominal fat deposition chickens at 1, 4, and 12 months (q-PCR). Scale bars = 50 μm. H represents high abdominal fat chickens (*n* = 10), and L represents low abdominal fat chickens (*n* = 10). Statistical significance between groups was determined by unpaired Student’s *t* tests. All data were presented as mean ± SEM. *p < 0.05.

**Fig. 4:**
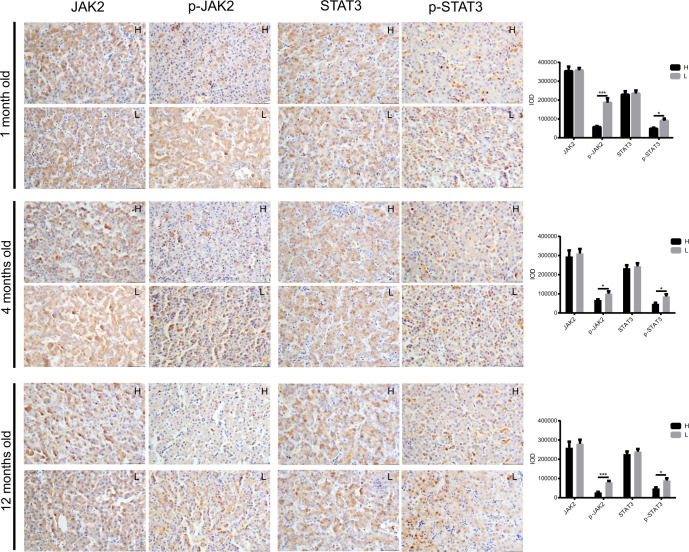
Comparing the expression of fat metabolism related proteins in liver between high and low abdominal fat deposition chickens at different months. The protein distribution and expression levels of JAK2, p-JAK2, STAT3, p-STAT3 in high and low abdominal fat deposition chickens at 1, 4, and 12 months old, respectively (IHC). Scale bars = 50 μm. H represents high abdominal fat chickens (*n* = 10), and L represents low abdominal fat chickens (*n* = 10). Statistical significance between groups was determined by unpaired Student’s *t* tests. All data were presented as mean ± SEM. *p < 0.05, ***p < 0.001.

It has been established that unbalanced fat metabolism is closely related to abdominal fat deposition, so the fat metabolism levels in the blood (TG, TC, LDL-C, and HDL-C), abdominal fat, and liver were compared between high and low abdominal fat deposition chickens at different ages (1 month old, 4 months old, and 12 months old). In blood, the concentrations of TG (4 months old: *p* = 0.0025), TC (12 months old: *p* = 0.0406), and LDL-C (1 month old: *p* = 0.0273, 12 months old: *p* = 0.0183) were markedly higher in high abdominal fat deposition chickens at some time points, yet the concentration of HDL-C (1 month old: *p* = 0.0436, 4 months old: *p* = 0.0392, 12 months old: *p* = 0.0483) was significantly higher in low abdominal fat deposition chickens at all time points (Fig. 2a). In abdominal fat, the relative mRNA expressions of some fat synthesis-related genes, such as *ACC*, *FAS*, and *LPL*, were markedly (unpaired Student’s *t* tests, *p* < 0.05) higher in high abdominal fat deposition chickens at all time points (Fig. 2b), yet the relative mRNA expression of fat catabolism-related gene hormone-sensitive lipase (*HSL*) was significantly (4 months old: *p* = 0.0131, 12 months old: *p* = 0.0197) higher in low abdominal fat deposition chickens at the age of 4 and 12 months (Fig. 2c). In the liver, the number of hollow vesicular fat was more in high abdominal fat deposition chickens (Fig. 3a). The relative mRNA expressions of fat synthesis-related genes including acyl-CoA synthetase long chain family member 1 (*ACSL1*), fatty acid desaturase 1 (*FADS1*), and cytochrome P450 2C45 (*CYP2C45*) were significantly higher in high abdominal fat deposition chickens at all time points (*p* < 0.05) (unpaired Student’s *t* tests, Fig. 3b). Yet the relative mRNA expression of fat transport-related gene apolipoprotein A-I (*APOAI*) (Fig. 3c) was significantly (1 month old: *p* = 0.0291, 4 months old: *p* = 0.0144, 12 months old: *p* = 0.0297) higher and fat catabolism-related genes including peroxisome proliferator activated receptor alpha (*PPARα*), carnitine palmitoyl transferase 1 (*CPT-1*), leptin receptor (*LEPR*), Janus kinase 2 (*JAK2*), and signal transducer and activator of transcription 3 (*STAT3*) was significantly (unpaired Student’s *t* tests, *p* < 0.05) higher in low abdominal fat deposition chickens at different time points (Fig. 3d). Furthermore, the protein expression levels of p-JAK2 (1 month old: *p* = 0.0005, 4 months old: *p* = 0.0345, 12 months old: *p* = 0. 0.00014) and p-STAT3 (1 month old: *p* = 0.0217, 4 months old: *p* = 0.0328, 12 months old: *p* = 0.0205) were significantly higher in low abdominal fat deposition chickens at all time points (Fig. 4).

### The cecal microbiota is significantly different between high and low abdominal fat deposition chickens

**Fig. 5:**
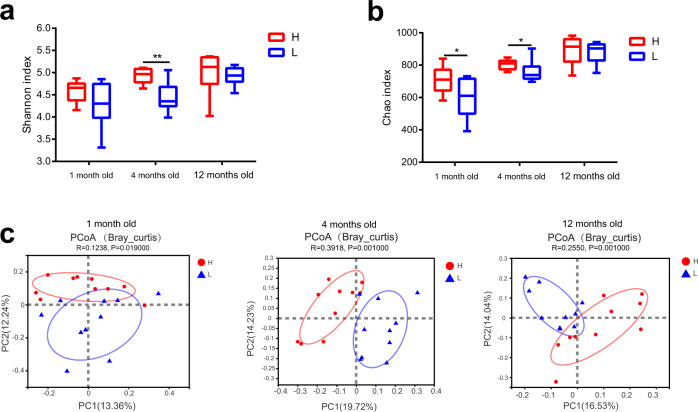
Comparison of microbial α and β diversity in cecum between high and low abdominal fat deposition chickens at different months. The comparison of microbial community diversity measured with the Shannon index **a** and Chao index **b** between high and low abdominal fat deposition chickens at different months. **c** The comparison of the principal co-ordinates analysis (PCoA) based on OTU between high and low abdominal fat deposition chickens at 1, 4, and 12 months. H represents high abdominal fat chickens (*n* = 10), and L represents low abdominal fat chickens (*n* = 10). The center line represents median, the bounds of box represent the first and third quartiles, and whisker shows the minimum and maximum values, and the statistical significance between the groups was determined by Wilcoxon rank-sum test (**a**, **b**). ANOSIM (Analysis of similarities) analysis is used to test whether the difference between groups (two or more groups) is significantly greater than the difference within the group, so as to judge whether the grouping is meaningful (**c**). *p < 0.05, **p < 0.01.

**Fig. 6:**
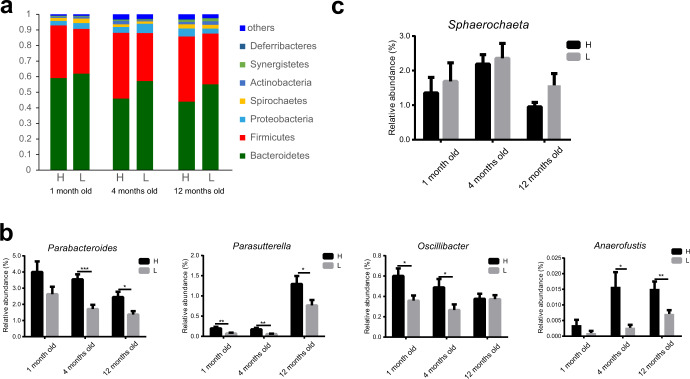
Analysis of microbial community composition and difference in cecum between high and low abdominal fat deposition chickens at different months. **a** Cecal microbiota community composition at the phylum level in chickens at different (1, 4, and 12) months. **b**, **c** The comparison of the relative abundance of target genera (*Parabacteroides*, *Parasutterella, Oscillibacter*, *Anaerofustis, Sphaerochaeta*) between high and low abdominal fat deposition chickens at different (1, 4, and 12) months, respectively. H represents high abdominal fat chickens (*n* = 10), and L represents low abdominal fat chickens (*n* = 10). Statistical significance between groups was determined by unpaired Student’s *t* tests (**b**, **c**). All data were presented as mean ± SEM. *p < 0.05 **p < 0.01, ***p < 0.001.

16S rRNA gene sequencing was used to compare the cecal microbiota composition between high and low abdominal fat deposition chickens at different time points. Alpha-diversity analysis indicated that the microbial diversity (Fig. 5a) and community abundance (Fig. 5b) in high abdominal fat deposition were higher than low abdominal fat deposition chickens. Beta-diversity exhibited distinct separation between high and low abdominal fat deposition chickens at different time points (ANOSIM analysis, *p* < 0.05; Fig. 5c). At the phylum level, Firmicutes were more abundant in high abdominal fat deposition chickens, while Bacteroidetes were more abundant in low abdominal fat deposition chickens at all time points (Fig. 6a). At the genus level, the relative abundance of *Parabacteroides* (4 months old: *p* = 0.0003, 12 months old: *p* = 0.0131), *Parasutterella* (1 month old: *p* = 0.0083, 4 months old: *p* = 0.0041, 12 months old: *p* = 0.0390), *Oscillibacter* (1 month old: *p* = 0.0134, 4 months old: *p* = 0.0384), and *Anaerofustis* (4 months old: *p* = 0.0137, 12 months old: *p* = 0.0079) was significantly higher in high abdominal fat deposition chickens (Fig. 6b), while the relative abundance of *Sphaerochaeta* was higher in low abdominal fat deposition chickens (Fig. 6c and Supplementary Fig. 2).

### Metagenomic analysis revealed distinct functional differences of the cecal microbiota between high and low abdominal fat deposition chickens

**Fig. 7:**
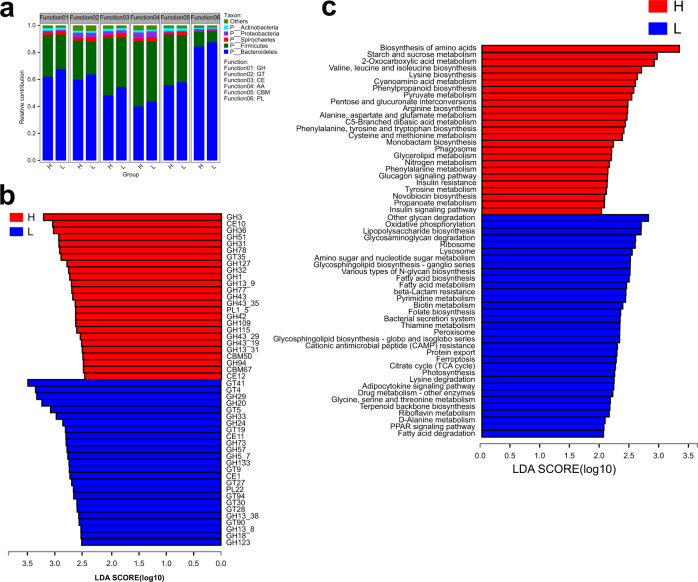
Analysis of functional differences of cecal microbiota between high and low abdominal fat deposition chickens at four months. **a** The comparison of the relative contribution of cecal microbiota (at phylum level) to carbohydrate active enzymes (CAZymes) between high and low abdominal fat deposition chickens. **b** The comparison of carbohydrate enzymatic activities of cecal microbiota between high and low abdominal fat deposition chickens. **c** The comparison of KEGG differential pathways of cecal microbiota between high and low abdominal fat deposition chickens. H represents high abdominal fat chickens (*n* = 10), and L represents low abdominal fat chickens (*n* = 10). LDA score (log10) > 2.0.

The association between cecal microbiota and carbohydrate-active enzymes (CAZymes) including glycoside hydrolases (GHs), glycosyltransferases (GTs), carbohydrate esterases (CEs), auxiliary activities (AAs), carbohydrate-binding modules (CBMs), and polysaccharide lyases (PLs) was analyzed. Firmicutes and Bacteroidetes encoded more than 85% of the main CAZymes. Compared with high abdominal fat deposition chickens, Firmicutes encoded fewer CAZymes, yet Bacteroidetes encoded more CAZymes in low abdominal fat deposition chickens (Fig. 7a). Further analysis indicated that 25 CAZymes were found higher counts in high abdominal fat deposition chickens, and 19 of them are GHs. Other 25 CAZymes were found higher counts in low abdominal fat deposition chickens, 12 are GHs, and 10 are GTs (Fig. 7b). KEGG analysis showed that differentially expressed genes were annotated to 58 different pathways. Carbohydrate metabolism pathways including starch and sucrose metabolism, pyruvate metabolism, pentose and glucuronate interconversion, C5 branched chain dibasic acid metabolism, and propanoate metabolism were found higher counts in high abdominal fat deposition chickens. Lipid metabolism pathways including fatty acid biosynthesis and fatty acid degradation were found higher counts in low abdominal fat deposition chickens (Fig. 7c).

### 16S rRNA gene sequencing revealed that cecal microbiota was differentially related to abdominal fat deposition in chickens

**Fig. 8:**
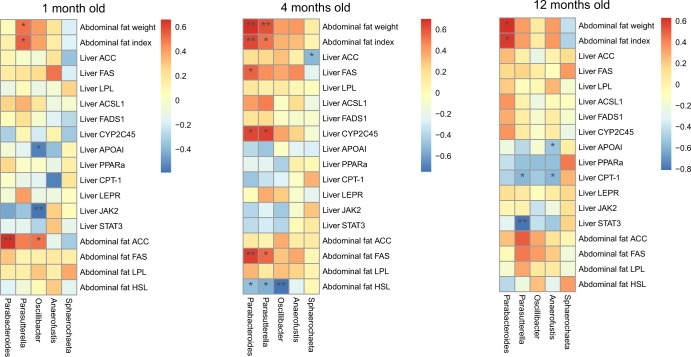
Correlation between cecum microbiota and abdominal fat deposition factors in high and low abdominal fat deposition chickens at different months. The association of different bacteria with the abdominal fat deposition and its related fat accumulation factors at 1, 4, and 12 months of age. Red color indicates positive correlation, and blue color indicates negative correlation. *p < 0.05, **p < 0.01.

Spearman correlation analysis was used to analyze the correlation between cecal microbiota and abdominal fat weight/index, and fat metabolism levels. The results indicated that the abundance of *Parabacteroides*, *Parasutterella*, *Oscillibacter*, and *Anaerofustis* was significantly (Spearman’s correlation tests, *p* < 0.05) and positively correlated with abdominal fat weight/index, and expression of fat synthesis-related genes in liver and abdominal fat, while significantly (Spearman’s correlation tests, *p* < 0.05) and negatively correlated with expression of fat transport and catabolism-related genes in liver and abdominal fat. Further, the abundance of *Sphaerochaeta* was positively correlated with the expression of fat transport and catabolism-related genes in liver and abdominal fat and negatively correlated with abdominal fat weight/index (Fig. 8).

### Fecal microbiota transplantation from high or low abdominal fat deposition chickens significantly changed fat deposition of recipients

**Fig. 9:**
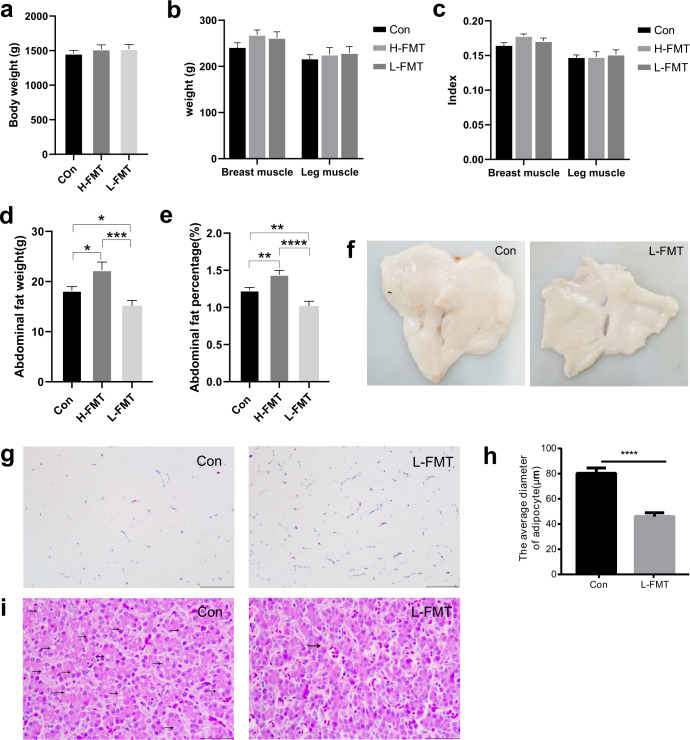
FMT altered the fat deposition of chickens. **a** An increasing trend of body weight in the H-FMT and L-FMT groups compared with the control group. **b** An increasing trend of breast and leg muscle weight in the H-FMT and L-FMT groups compared with the control group. **c** An increasing trend of breast and leg muscle index in the H-FMT and L-FMT groups compared with the control group. **d** The comparison of abdominal fat weight between the control, H-FMT and L-FMT groups. **e** The comparison of abdominal fat index between the control, H-FMT and L-FMT groups. **f** The comparison of abdominal fat tissues of chickens between the control and L-FMT groups. **g**, **h** HE staining sections of abdominal adipose tissues and the comparison of an average diameter of adipocytes between the control and L-FMT groups. **i** HE staining sections of fat content in hepatocytes of the chickens. Scale bars (**g**) = 100 μm, Scale bars (**i**) = 50 μm. Con represents the control group (*n* = 30), H-FMT represents fecal microbiota transplantation group from the high abdominal fat deposition chicken (*n* = 30), L-FMT represents fecal microbiota transplantation group from the low abdominal fat deposition chicken (*n* = 30). Statistical significance between groups was determined by unpaired Student’s *t* tests. All data were presented as mean ± SEM. *p < 0.05, **p < 0.01, ***p < 0.001, ****p < 0.0001.

In order to verify the effects of gut microbiota on chicken abdominal fat deposition, the fecal microbiota from adult chickens with high or low abdominal fat deposition was transplanted into 1-day-old chicks. The increasing trends of body weight (Fig. 9a), breast/leg muscle weight (Fig. 9b), and breast/leg muscle index (Fig. 9c) were observed in the FMT groups compared with the control group. Four weeks of FMT from high abdominal fat deposition chicken significantly increased abdominal fat weight (H-FMT: 22.29 ± 1.59 g vs Con: 18.19 ± 0.79 g) (unpaired Student’s *t* tests, *p* = 0.0286) (Fig. 9d) and abdominal fat index (H-FMT: 1.44 ± 0.06% vs Con: 1.23 ± 0.04%) (unpaired Student’s *t* tests, *p* = 0.0050) (Fig. 9e). Interestingly, four weeks of FMT from low abdominal fat deposition chicken significantly decreased abdominal fat weight (L-FMT: 15.18 ± 1.05 g vs Con: 18.19 ± 0.79 g) (unpaired Student’s *t* tests, *p* = 0.0278) (Fig. 9d) and abdominal fat index (L-FMT: 1.02 ± 0.06% vs Con: 1.23 ± 0.04%) (unpaired Student’s *t* tests, *p* = 0.0072) (Fig. 9e). Furthermore, L-FMT decreased abdominal fat volume (Fig. 9f). HE staining results indicated that the average diameter of abdominal adipocytes was significantly lower in L-FMT group than that of control group (unpaired Student’s *t* tests, *p* < 0.0001) (Fig. 9g, h) and the number of hollow vesicular fat in liver was markedly less in L-FMT group (Fig. 9i). The above results indicated that FMT could significantly change chicken fat deposition.

### Fecal microbiota transplantation from low abdominal fat deposition chicken significantly modulated fat metabolism levels of recipients

**Fig. 10:**
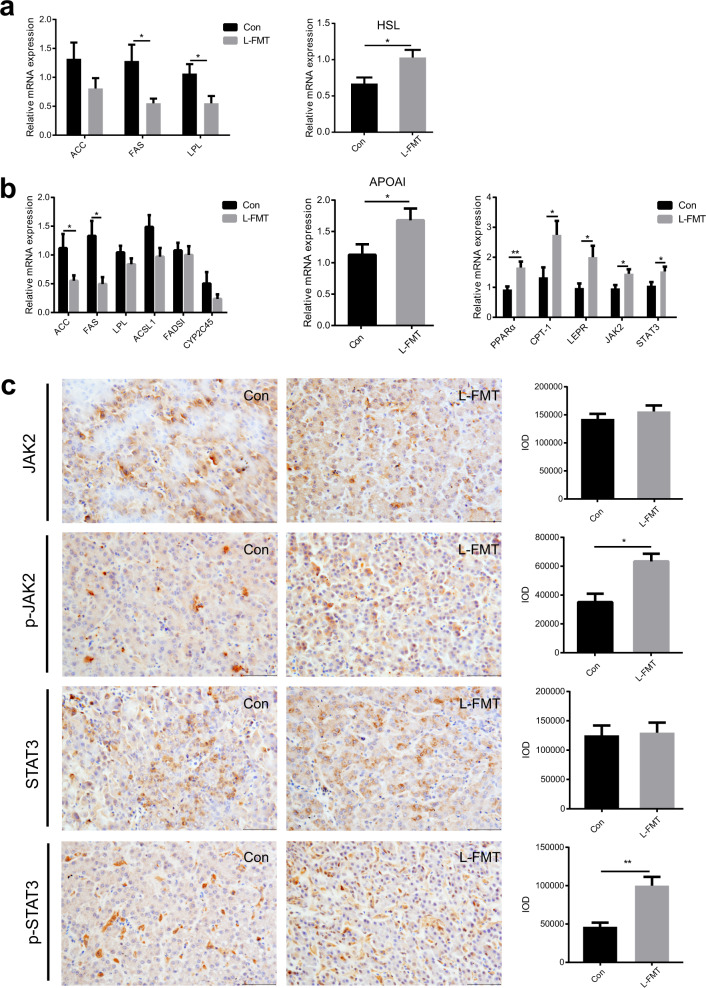
FMT changed the fat metabolism in abdominal fat and liver of chickens. The comparison of fat metabolism levels in the chicken abdominal fat and liver between the control and the L-FMT groups. **a** The comparison of relative mRNA expression of fat synthesis related genes and fat catabolism related genes in abdominal fat (q-PCR). **b** The comparison of relative mRNA expression of fat synthesis related genes, fat transport related genes, and fat catabolism related genes in the liver (q-PCR). **c** The protein distribution and the expression levels of JAK2, p-JAK2, STAT3, and p-STAT3 in the liver (IHC). Scale bars = 50 μm. Con represents the control group (*n* = 14), and L-FMT represents the fecal microbiota transplantation group from the low abdominal fat deposition chicken (*n* = 14). Statistical significance between groups was determined by unpaired Student’s *t* tests. All data were presented as mean ± SEM. *p < 0.05, **p < 0.01.

In order to verify the effects of L-FMT on the fat metabolism of recipients, the fat metabolism levels in abdominal fat and liver were investigated. In abdominal fat, qPCR results showed that L-FMT significantly down-regulated the relative mRNA expression of fat synthesis-related genes (*FAS* (unpaired Student’s *t* tests, *p* = 0.0313) and *LPL* (unpaired Student’s *t* tests, *p* = 0.0283)), and up-regulated the relative mRNA expression of *HSL* (unpaired Student’s *t* tests, *p* = 0.0283) (Fig. 10a). In liver, qPCR results showed that L-FMT significantly down-regulated the relative mRNA expression of fat synthesis-related genes, *ACC* (unpaired Student’s *t* tests, *p* = 0.0429), *FAS* (unpaired Student’s *t* tests, *p* = 0.0192)), and significantly up-regulated the relative mRNA expression of *APOAI* (unpaired Student’s *t* tests, *p* = 0.0422) and fat catabolism-related genes (*PPARα*, *CPT-1*, *LEPR*, *JAK2*, and *STAT3*) (unpaired Student’s *t* tests, *p* < 0.05) (Fig. 10b). Immunohistochemistry (IHC) staining results indicated that L-FMT significantly up-regulated protein expression of p-JAK2 (unpaired Student’s *t* tests, *p* = 0.0115) and p-STAT3 (unpaired Student’s *t* tests, *p* = 0.0055) in liver (Fig. 10c).

### Fecal microbiota transplantation from low abdominal fat deposition chicken reshaped the cecal microbiota of the recipients

**Fig. 11:**
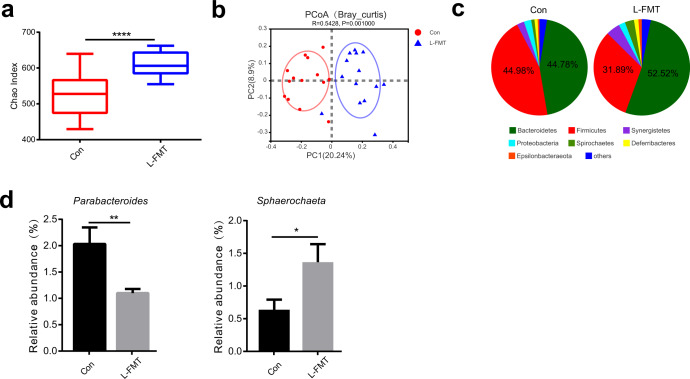
FMT changed the composition of chicken cecal microbiota. For 16S rRNA sequencing, 28 white feather broilers (14 in each group with equal males and females) were randomly selected for cecal microbiota analysis in the control and L-FMT groups. **a** The comparison of Chao index. **b** Principal co-ordinates analysis (PCoA) analysis. **c** Cecal microbiota community composition at the phylum level. **d** The relative abundance of *Parabacteroides* and *Sphaerochaeta*. Con represents the control group (*n* = 14), and L-FMT represents the fecal microbiota transplantation group from the low abdominal fat deposition chicken (*n* = 14). The center line represents median, the bounds of box represent the first and third quartiles, whisker shows the minimum and maximum values and statistical significance between groups was determined by Wilcoxon rank-sum test (**a**). ANOSIM (Analysis of similarities) analysis is used to test whether the difference between groups (two or more groups) is significantly greater than the difference within the group, so as to judge whether the grouping is meaningful (**b**). Statistical significance between groups was determined by unpaired Student’s *t* tests and the data were presented as mean ± SEM (**d**). *p < 0.05, **p < 0.01, ****p < 0.0001.

16 S rRNA gene sequencing results showed that L-FMT significantly increased cecal microbial community abundance (Chao index) (Wilcoxon rank-sum test, *p* < 0.0001) (Fig. 11a), changed cecal microbiota composition (ANOSIM analysis, *p* < 0.001) (Fig. 11b), increased relative abundance of Bacteroidetes, and decreased relative abundance of Firmicutes (Fig. 11c). Further, L-FMT significantly (unpaired Student’s *t* tests, *p* = 0.0078) decreased the relative abundance of *Parabacteroides* and significantly (unpaired Student’s *t* tests, *p* = 0.0298) increased the relative abundance of *Sphaerochaeta* compared with the control group (Fig. 11d and Supplementary Fig. 3).

## Discussion

Stable fat metabolism liberates energy to increase growth, yet unstable fat metabolism often results in an unnecessary fat deposition^23^. Fat metabolism is a complex biochemical mechanism in which fat digestion, assimilation, and transportation occur through several anabolic and catabolic reactions^21^. The digested fat is further processed in the form of fatty acids and glycerol^21^. For instance, the produced fatty acids and glycerol are absorbed in the intestinal epithelium and transported through blood circulation to the liver, adipose tissues, and other organs^21^. Fat synthesis in organs is regulated by fat synthesis-related genes, including *FAS*, *ACSL1*, *FADS1*, *CYP2C45*, and *LPL*^24^. To understand the impact of the fat anabolism pathway on fat metabolism, the expression of fat anabolism-related genes was elucidated. In the present study, a significantly higher relative mRNA expression of *FAS, ACSL1, FADS1, CYP2C45*, and *LPL* was found in liver and abdominal fat of high abdominal fat deposition chickens, suggesting more fat synthesis, which would have close connection with the fat deposition in chickens. It has also been reported that elevated *ACSL1, ACC*, and *FAS* are associated with fat deposition through increasing serum TG, TC, and LDL-C levels^25,26^. In the present study, the increased serum TG, TC, and LDL-C levels and significantly decreased serum HDL-C levels in high abdominal fat deposition chickens are consistent with the findings in mice^27^, and in chickens^28,29^, indicating that deposition of a higher amount of abdominal fat would also alter TG, TC, HDL-C, and LDL-C levels in chicken serum. Further, it has also been demonstrated that fat catabolism genes significantly contribute to the fat metabolism. For instance, *CPT-1* and *PPARα* are catabolic genes that stimulate fatty acid’s oxidation, resulting in energy production for chicken growth^30^. In the present study, a significant downregulated hepatic mRNA expression of *CPT-1* and *PPARα* and a lower JAK2 expression via STAT3 activation in high abdominal fat deposition chickens remarkably increased abdominal fat deposition in broilers^31,32^. Notably, elevated *APOAI* and *HSL* and higher serum HDL-C levels in low abdominal fat deposition chickens in our study is predicted to facilitate fat excretion because *HSL* acts as a cleaner and, along with *APOAI*, transports cholesterol/fatty acids from fat depots to liver for lipolysis, reducing fat accumulation in broilers^33–35^. Additionally, upregulated adipocyte differentiation-related genes increase their proliferation and significantly contribute to abdominal fat deposition in chickens^5,21^. A significantly higher average diameter of abdominal adipocytes in high abdominal fat deposition chickens in our study, indicates the crucial role of adipocytes differentiation in fat deposition^36^. Therefore, more fat synthesis and less fat catabolism resulted in excessive fat deposition and vice versa.

It has been established that gut microbiota could control abdominal fat deposition by regulating fat metabolism^6^. For example, the abundance of *Parabacteroides* is positively correlated with fat mass in obese individuals^37,38^. *Parasutterella* causes irritable bowel syndrome and immunosuppression in chickens^39^, and increases abdominal fat percentage/deposition^40^. *Oscillibacter* is abundant in fat-line chickens^41^, and is associated with obesity^42^. Likewise, an increased abundance of *Anaerofustis* in the cecum of high-fat diet mice^43^, and of broilers during *Clostridium perfringens*-induced infection^44^, is linked with fat metabolism. Recent studies revealed that the above-described bacteria closely interact with fat metabolism related genes and TG, TC, LDL-C, and HDL-C parameters to alter fat deposition. For instance, Li et al. described that *Parabacteroides* and *Oscillibacter* are positively related to FAS and ACC, while negatively related to HSL^45^. Similarly, both of these bacteria contribute to increasing hepatic TG and serum TG, TC, and LDL-C levels in high-fat diet mice^45^, indicating more fat anabolism rather than fat catabolism, which is predicted to increase fat deposition. Further, Huang et al. found that *Parasutterella* is involved in increasing TG, TC, and LDL-C levels, while decreasing HDL-C levels both in the liver and serum of mice^46^, and its higher abundance increases abdominal fat deposition because *Parasutterella* could break down high-energy foods and alter fat metabolism^40,47^. Another recent study reported that *Anaerofustis* could also regulate fat metabolism as it is positively associated with TG, TC, and LDL-C levels and negatively with HDL-C levels in serum^48^. Typically, these bacteria have a significant contribution to fat accumulation and are consistent with our findings of high abdominal fat deposition chickens, indicating that higher abundances of these bacteria in the host gut could increase fat deposition. Interestingly, some other bacteria have also been found that behave differently as compared to those described above. For instance, *Sphaerochaeta* is importantly related to decreasing fat deposition in swine^49^, and is recognized as a core species to regulate lipid metabolism^50^. A recent study found that *Sphaerochaeta* is significantly enriched in lean chickens^14^, which is also consistent with our findings. Further, Huang et al. reported that *Sphaerochaeta* is positively linked with serum HDL-C level and negatively with TC level and could improve fat metabolism^51^. Feng et al. also described that a higher abundance of *Sphaerochaeta* remarkably regulates body fat metabolism and could control abdominal fat deposition in Xianju yellow chicken^52^, indicating *Sphaerochaeta* could affect fat metabolism and fat deposition. Therefore, higher abundances of *Parabacteroides*, *Parasutterella*, *Oscillibacter*, and *Anaerofustis* are predicted to an increased fat deposition trend in high abdominal fat deposition chickens through fat anabolism, whereas higher abundance of *Sphaerochaeta* is expected to the decreased fat deposition trend in low abdominal fat deposition chickens through fat catabolism.

It has been established that gut microbiota could encode CAZymes to regulate fat deposition^14,53^. Gut microbiota mainly degrades resistant starch and dietary fibers through hydrolysis^14,53^ and accomplishes this function through carbohydrate metabolism by using GHs, GTs, CEs, PLs, and CBMs^54,55^. Typically, carbohydrate metabolism is interlinked with fat deposition because higher calories produced by carbohydrate metabolism might cause de novo lipogenesis and a huge conversion of glucose into pyruvate (glycolysis) or into TG^23,42,56^. It has been reported that *Oscillibacter* has high number of GHs and CBMs genes to cleave the complex polysaccharides and could also regulate fat deposition^41,57,58^. In the present study, both *Anaerofustis* and *Oscillibacter* (Firmicutes) are found significantly enriched in high abdominal fat deposition chickens, predicting that these bacteria extracted extra energy by using CAZymes and transported it to adipose tissues, resulting in excessive abdominal fat deposition. Other studies also found a positive association of *Anaerofustis* and *Oscillibacter* with fiber digestibility and obesity^59,60^. On the other hand, Xiang et al. found that *Sphaerochaeta* could considerably promote CAZyme activities and regulate lipid metabolism in the lean chickens^14^, which is in accordance with our findings that *Sphaerochaeta* as a unique member could decrease abdominal fat deposition through CAZymes (GHs, GTs, CEs, AA, and CBMs) activities. Evidence reveals that *Sphaerochaeta* belongs to phylum Spirochaetes^61^ and several studies described that *Sphaerochaeta* could also produce β-xylosidases of GH family, process carbohydrate polymers through carbohydrate metabolism, and had significant contribution to regulate fat deposition^50,62,63^. Thus, it is anticipated that *Sphaerochaeta* has significant potential in controlling abdominal fat deposition through encoding CAZymes.

Increasing pieces of evidence indicated that reshaping gut microbiota by fecal microbiota transplantation (FMT) could reduce abdominal fat deposition by regulating fat metabolism^14,64^. Consistent with these findings, the present study also observed that FMT from the low abdominal fat deposition chicken significantly reduced abdominal fat deposition (both abdominal fat weight and index) and remarkably regulated fat metabolism. Further, it was reported that *Sphaerochaeta* was enriched in lean chickens, and *Parabacteroides* was enriched in obese individuals^14,37^. Likewise, a higher abundance of *Sphaerochaeta* and a lower abundance of *Parabacteroides* were observed in the L-FMT group of the present study, which are consistent with our results in low abdominal fat deposition chickens, indicating that these bacteria could alter abdominal fat deposition and control fat metabolism. Other studies also described that FMT could significantly attenuate fat deposition in high fat diet mice^65^ because lower LPL level decreases adipogenesis by reducing triglyceride hydrolysis in the adipose tissues^66^. In the present study, significantly decreased expressions of anabolism related genes (*FAS*, *LPL* in abdominal fat and *ACC*, *FAS* in liver), and significantly increased expressions of catabolism related genes (hepatic *PPARα, CPT-1, LEPR, JAK2*, and *STAT3*) were found in the L-FMT group compared with the control. Furthermore, a significantly higher expression of *HSL* in abdominal fat and *APOAI*, *p-JAK2*, and *p-STAT3* in liver of the L-FMT group was also observed. The results indicated that fecal microbiota from low abdominal fat deposition chickens could increase the abundance of *Sphaerochaeta*, which promoted fat catabolism by enhancing the expressions of fat catabolic and fat transport-related genes^67^.

## Conclusion

Taken together, the current findings indicated that the unbalanced fat metabolism leads to excessive abdominal fat deposition. The abundances of *Parabacteroides*, *Parasutterella*, *Oscillibacter* and *Anaerofus* are correlated with upregulating expression of fat anabolism genes, which eventually increases abdominal fat deposition. However, the abundances of *Sphaerochaeta* upregulates the expression of fat catabolism genes, which reduces abdominal fat deposition and benefits the muscle growth of the chickens. Moreover, L-FMT significantly decreased *Parabacteroides*, increased *Sphaerochaeta*, and upregulated the expression of fat catabolism genes. L-FMT might be applied as a strategy in reducing abdominal fat deposition and at the same time promoting the growth of muscles.

## Methods

### Animals

The Institutional Animal Care and Use Committee of Huazhong Agricultural University (HZAUCH-2018-008), Wuhan, China approved all the animal procedures, and all methods were performed in accordance with the relevant guidelines and regulations.

Newly hatched chickens (Turpan cockfighting × White Leghorn) were reared under similar husbandry conditions on the chicken farm of Huazhong Agricultural University. At the age of 1, 4, and 12 months, 120 chickens were randomly selected for each time point. Based on the abdominal fat index, the chickens at each time point were categorized into two groups, namely the high abdominal fat deposition group (H) and the low abdominal fat deposition group (L) (*n* = 10, 5 males and 5 females). For the FMT experiment, the chickens with high body weight, low abdominal fat deposition and high abdominal fat deposition were separately selected as FMT donors. A total of 90 one-day-old white feather broilers were selected as recipients.

### Selection of FMT donors

Two adult female white Leghorn chicken × Turpan fighting chickens possibly having high or low abdominal fat deposition were scanned with computed tomography (CT) instrument (Aquilion PRIME Tsx-303A, Canon Medical, Japan). Pari software was used to mark the abdominal fat in different frame images of each chicken (Supplementary Fig. 1), and then Python language was used to write programs to analyze the images and calculate the volume of the body and abdominal fat of each chicken. The volume of the body was 2.22 dm^3^ and 2.50 dm^3^, and the volume of abdominal fat was 0.06 dm^3^ and 0.15 dm^3^, respectively. Similarly, the volume percentage of abdominal fat was 2.66% and 5.92%. The chicken with less abdominal fat volume percentage was selected as L-FMT group donor and the chicken with more abdominal fat volume percentage was selected as H-FMT group donor. After FMT experiment, the two chickens were dissected to get the abdominal fat weight and index. The abdominal fat weight was 74.3 g and 161.2 g, and the abdominal fat index was 3.12% and 5.78%, respectively, which are consistent with the CT results and indicated that the FMT donor selection is appropriate.

### Preparation of fecal suspension

Every morning, once the donor chickens defecated, the white part of the fecal materials was removed as it contains uric acid. Then 10 g of feces were collected in the sterile tube (50 mL) and gently mixed with 60 mL of 0.75% normal saline. The mixture was kept on the ice for settling down the precipitates. The supernatant was obtained and filtered with sterile gauze to get fecal suspension.

### Animal treatment

A total of ninety 1-day-old white feather broilers were selected as recipients and randomly divided into control group, H-FMT group and L-FMT group (*n* = 30). The chickens were fed a corn-soybean diet in pellet form with no medication or vaccination. All chickens were kept in the same room. From the 1st day till the end of the experiment, 2 chickens in each cage (length = 70 cm, width = 50 cm, and height = 60 cm) were kept. Broilers in FMT group were orally administrated with 1 mL fecal microbiota suspension, while 1 mL 0.75% saline was used as a substitute in the control group for 28 days. At the age of 42 days, the birds were euthanized by gradually increasing CO_2_ inhalation for about 4–5 min time period, then humanely killed by puncturing the jugular vein and collecting the blood sample at the same time. Subsequently, the other samples were collected^68^.

### Sample collection

After fasting for 12 h, the chickens were weighed and killed, then blood (through the jugular vein), liver, abdominal adipose tissue, and left cecum were collected. For gut microbiota analysis, the cecal content (1–1.5 g per bird) was collected into two sterilized centrifuge tubes (1.5 mL) and snap-frozen in liquid nitrogen, then stored at −80 °C for sequencing. For analysis of lipometabolic parameter, blood samples (3 mL per bird) were centrifuged at 3000 × *g* at 4 °C for 15 min to get the serum, and then it was stored at −80 °C for subsequent analysis. For histo-morphological analysis, freshly harvested liver and abdominal adipose tissues were fixed in 4% paraformaldehyde solution. For gene expression analysis, parts of freshly harvested liver and abdominal adipose tissues were snap-frozen in liquid nitrogen and then stored at −80 °C.

### Muscle or abdominal fat index calculation

The muscle or abdominal fat index was calculated using the following formula: muscle index = muscle weight (g)/body weight (g) × 100%, abdominal fat index = abdominal fat weight (g)/ body weight (g) × 100%.

### 16S rRNA and Metagenomic genes sequencing

Microbial genomic DNA was extracted from the chicken’s cecal content using Fast DNA SPIN extraction kit (MP Biomedicals, Santa Ana, CA, USA), according to manufacturer’s instructions. The hypervariable region V3-V4 of the bacterial 16S rRNA gene was amplified with primer pairs 338F (5′-ACTCCTACGGGAGGCAGCAG-3′) and 806R (5′-GGACTACHVGGGTWTCTAAT-3′). The PCR amplification of the 16S rRNA gene was performed as follows: an initial denaturation (3 min) at 95 °C following 27 cycles of denaturing (30 s) at 95 °C, annealing (30 s) at 55 °C, extension (45 s) at 72 °C, and single extension (10 min) at 72 °C, and ended at 4 °C. The PCR product was extracted from 2% agarose gel and purified using the AxyPrep DNA Gel Extraction Kit (Axygen Biosciences, Union City, CA, USA) according to manufacturer’s instructions and quantified using Quantus™ Fluorometer (Promega, USA). Illumina MiSeq PE300 platform (Illumina, San Diego, USA) was used for 16S rRNA gene sequencing. For 20 chickens at the age of 4 months with metagenomic sequencing, the same DNA extract was fragmented to an average size of about 400 bp using Covaris M220 (Gene Company Limited, China) for paired-end library construction, which was constructed using NEXTFLEX Rapid DNA-Seq (Bioo Scientific, Austin, TX, USA). Illumina NovaSeq platform (Illumina, San Diego, CA, USA) was used for metagenomic sequencing.

### 16S rRNA gene sequencing data processing

The raw 16S rRNA gene sequencing reads were demultiplexed, quality-filtered by fastp version 0.20.0, and merged by FLASH version 1.2.7. Operational taxonomic units (OTUs) with 97% similarity cutoff were clustered using UPARSE version 7.1, and chimeric sequences were identified and removed. The taxonomy of each OTU representative sequence was analyzed by RDP Classifier version 2.2 against the 16S rRNA database (Silva 132) using a confidence threshold of 0.7. For α and β diversity measurements, the sequencing depth was minimized by subsampling the readings of each sample. The lowest valid reads of cecal microbiota of high and low abdominal fat deposition chickens at the age of 1 month were 25,339, the lowest effective reading of cecal microbiota of high and low abdominal fat deposition chickens at the age of 4 months was 30,671, and the lowest effective reading of cecal microbiota of high and low abdominal fat deposition chickens at the age of 12 months was 45,053. Similarly, the lowest valid reads of cecal microbiota in the control and L-FMT chickens were 14,960. The α-diversity was described using the Shannon index and Chao index. Principal coordinates analysis (PCoA) based on Bray–Curtis was used to estimate the dissimilarity in the community structure. The community composition at the phylum level and the change of abundance at the genus level were visualized by bar chart and histogram. Linear discriminant analysis effect size (LEfSe) was performed to detect differentially abundant taxa across groups using the default parameters linear discriminant analysis (LDA > 2).

### Metagenomic sequencing data processing

The low-quality reads (length <50 bp or with a quality value < 20 or having N bases) were removed by fastp (https://github.com/OpenGene/fastp, version 0.20.0). Reads were aligned to the chicken genome by burrows-wheeler alignment (BWA) tool (http://bio-bwa.sourceforge.net, version 0.7.9a), and any hit associated with the reads and their mated reads were removed. The optimized sequence was spliced and assembled, and contigs ≥300 bp were selected as the final assembly result, and then the contigs were used for further gene prediction and annotation. Open reading frames (ORFs) from each assembled contig were predicted using MetaGene (http://metagene.cb.k.u-tokyo.ac.jp/). The predicted ORFs with length ≥100 bp were retrieved and translated into amino acid sequences. A non-redundant gene catalog was constructed using CD-HIT (http://www.bioinformatics.org/cd-hit/, version 4.6.1) with 90% sequence identity and 90% coverage. Reads after quality control were mapped to the non-redundant gene catalog with 95% identity using SOAPaligner (http://soap.genomics.org.cn/, version 2.21), and gene abundance in each sample was evaluated. Public data used for taxonomic analysis and gene functional classification included the integrated NCBI-NR database, KEGG database, and CAZy database. The amino acid sequence of non-redundant gene was aligned to NR database and KEGG database respectively with an e-value cutoff of 1e^−5^ using Diamond (http://www.diamondsearch.org/index.php, version 0.8.35), and obtained the species annotation and KEGG function corresponding to the gene. Carbohydrate-active enzymes annotation was conducted using hmmscan (http://hmmer.janelia.org/search/hmmscan) against the CAZy database (http://www.cazy.org/) with an e-value cutoff of 1e^−5^.

### Blood parameters analysis

For the analysis of different blood parameters, the serum concentrations of triglycerides (TG), total cholesterol (TC), high density lipoprotein cholesterol (HDL-C), and low density lipoprotein cholesterol (LDL-C) were determined using a Rayto Chemistry Analyzer (Chemray 800, China) according to the manufacturer’s instructions (Shenzhen Rayto Life Science Co., Ltd). Briefly, the serum samples were thoroughly mixed with the reaction solution in the recommended proportion and maintained at 37 °C for 10 min. Finally, the absorbance for each sample was measured, and the total concentrations were calculated according to the following formula. Total concentrations = Absorbance of sample/Absorbance of calibration solution × Calibration concentrations (mmol per liter).

### Hematoxylin and eosin staining

For morphological observation, liver and abdominal fat tissue samples were embedded in paraffin, and sections were prepared. Liver tissues were cut into 3 µm thick sections, and abdominal fat tissues were cut into 7 µm thick sections with a rotary slicer (LEICA 819, Leica, Germany). HE staining was performed according to the routine protocol, and stained tissue sections were examined with light microscope (BH-2, Olympus, Japan) using a digital camera (DP72, Olympus, Japan). Under 10 × 20 microscope, every HE stained section of abdominal fat was used to randomly select 5 visual fields for the image acquisition. The average diameter of abdominal fat adipocytes was measured with image pro plus 6.0 (Media Cybernetics, USA).

### Real-time quantitative polymerase chain reaction

In order to detect the expression of fat metabolism-related genes on mRNA level, total RNA was extracted from abdominal adipose and liver tissues using Trizol reagent (Takara, Japan) following the instructions of the manufacturer. RNA (1 μg) from each sample was reverse transcribed into cDNA using the PrimeScript™ RT reagent Kit with gDNA Eraser (Takara, Japan). The quantitative polymerase chain reaction (qPCR) reaction mixture (10 μL) consisted of 5 μL of SYBR (Takara, Japan), 0.4 μL of forward and 0.4 μL of reverse primer, 3.2 μL of ddH_2_O, and 1 μL of template cDNA. The qPCR reaction is carried out on Bio-Rad CFX Connect real-time qPCR detection system (Bio-Rad, Hercules, CA, USA). The steps are as follows: 5 min pre-denaturation at 95 °C, following 30 s denaturation at 95 °C (40 cycles), 30 s annealing at 60 °C, and 15 s elongation at 72 °C. The sequences of primers were listed in Table 1 with reference gene (*β-actin*). Gene expression levels were quantified using the 2^−ΔΔCT^ method.

**Table 1:** Primers used for real-time qPCR.

Gene	Forward Sequence (5′-3′)	Reverse Sequence (5′-3′)	Gene Bank No.
*β-actin*	TTGTTGACAATGGCTCCGGT	TCTGGGCTTCATCACCAACG	NM_205518.2
*ACC*	TCCAGCAGAACCGCATTGACAC	GTATGAGCAGGCAGGACTTGGC	NM_205505.2
*FAS*	GCTCTGCGTCTGCTTCAGTCTAC	GGTACAGGACTCTGCCATCAATGC	NM_205155.4
*LPL*	TGGACATTGGTGACCTGCTTATGC	TCGCCTGACTTCACTCTGACTCTC	NM_205282.2
*ACSL1*	GACTAATGGTCACAGGAGCAGCAC	CCAGGCATTGACAGTGAGCATCC	NM_001012578.2
*FADS1*	CCGTGCCACTGTGGAGAAGATG	GCCTAGAAGCAACGCAGAGAAGAG	XM_040673219.1
*CYP2C45*	AACAAGCACCACCACACGATACG	GGTCAGCCACGCAAGGTCTTC	NM_001001752.3
*APOAI*	GTGACCCTCGCTGTGCTCTT	CACTCAGCGTGTCCAGGTTGT	NM_205525.5
*PPARα*	TGCTGTGGAGATCGTCCTGGTC	CTGTGACAAGTTGCCGGAGGTC	XM_040699549.1
*CPT-1*	ACAGCGAATGAAAGCAGGGT	GCCATGGCTAAGGTTTTCGT	NM_001012898.1
*LEPR*	CACTCGCTGGGAACACTTGA	TTCAGCAGCCCATCGTTTCT	NM_204323.2
*JAK2*	GAGCGTGAGAATGCCACTGAC	TGGAGGACAGCACTTGATGAAC	NM_001030538.3
*STAT3*	GCCGAATCACAACTACAGACTC	CTGACTTTGGTGGTGAACTGC	NM_001030931.3
*HSL*	GAGGCACAGCGTCTTCTTTAGG	GGCACGAACTGGAACCCGAG	XM_040695201.1

### Immunohistochemistry

Following the steps described in earlier studies^13^, immunohistochemical staining was performed to observe the protein distribution and expression in the liver. Briefly, the sections were dewaxed twice in xylene and rehydrated in graded series ethanol. The antigen was retrieved in sodium citrate buffer (pH 6.0) using a microwave oven (MYA-2270M, Haier, Qingdao, China) for 18 min, i.e., 3 min at 700 W and fifteen min at 116 W, and then cooled for 2–3 h at room temperature. Endogenous peroxidase was inactivated with 3% hydrogen peroxide (H_2_O_2_), and tissue sections were incubated with 5% bovine serum albumin (BSA) (boster, China) at 37 °C for 30 min to block nonspecific binding sites. Then, the sections were incubated with primary antibodies of rabbit anti-JAK2 (1:100) (A11497, ABclonal Technology, Wuhan, China), rabbit anti-p-JAK2 (1:100) (AP0531, ABclonal Technology, Wuhan, China), rabbit anti-STAT-3 (1:100) (A1192, ABclonal Technology, Wuhan, China) and rabbit anti-p-STAT3 (1:100) (AP0474, ABclonal Technology, Wuhan, China). Subsequently, the horseradish peroxidase (HRP)-conjugated secondary antibody (Proteintech, China) was used to incubate the tissue sections for 30 min at 37 °C. After diaminobenzidine (DAB) (Proteintech, China) staining, the sections were counterstained with hematoxylin, cleaned and dehydrated until they became transparent, and finally sealed with neutral gum and coverslips. Finally, we used a light microscope (BH-2, Olympus, Japan) with a digital camera (DP72, Olympus, Japan) to examine the sections. Under a 10 × 40 microscope, every immunohistochemical section of liver was used to randomly select five positive visual fields for the image acquisition.

### Statistical analysis

Image Pro Plus 6.0 was used to calculate the integral optical density of positive signals. GraphPad Prism 6.0 (Media Cybernetics, USA) was used to analyze the test data. The measurement data were expressed as mean ± standard error of the mean (mean ± SEM). Statistical significance between groups was determined by unpaired Student’s *t* tests. A value of *p* < 0.05 was considered statistically significant.

## Supplementary information

Supplementary Figures

## Data Availability

The raw 16S rRNA gene and metagenomic sequencing data are available at the NCBI Sequence Read Archive (SRA), under BioProject PRJNA837471.

## Code availability

The code used for calculating the volume of the chicken body and abdominal fat in computed tomography (CT) figure is available at https://github.com/lyangfan/chicken-body-composition-calcaulation.
