# Supplementary material for: Chicken cecal microbiota reduces abdominal fat deposition by regulating fat metabolism
Source: NPJ Biofilms Microbiomes. 2023 May 30;9:28. doi: 10.1038/s41522-023-00390-8 (PMC10229630; doi:10.1038/s41522-023-00390-8)
Supplement: Supplementary file 1 — Supplementary Figures [file 41522_2023_390_MOESM1_ESM.pdf]

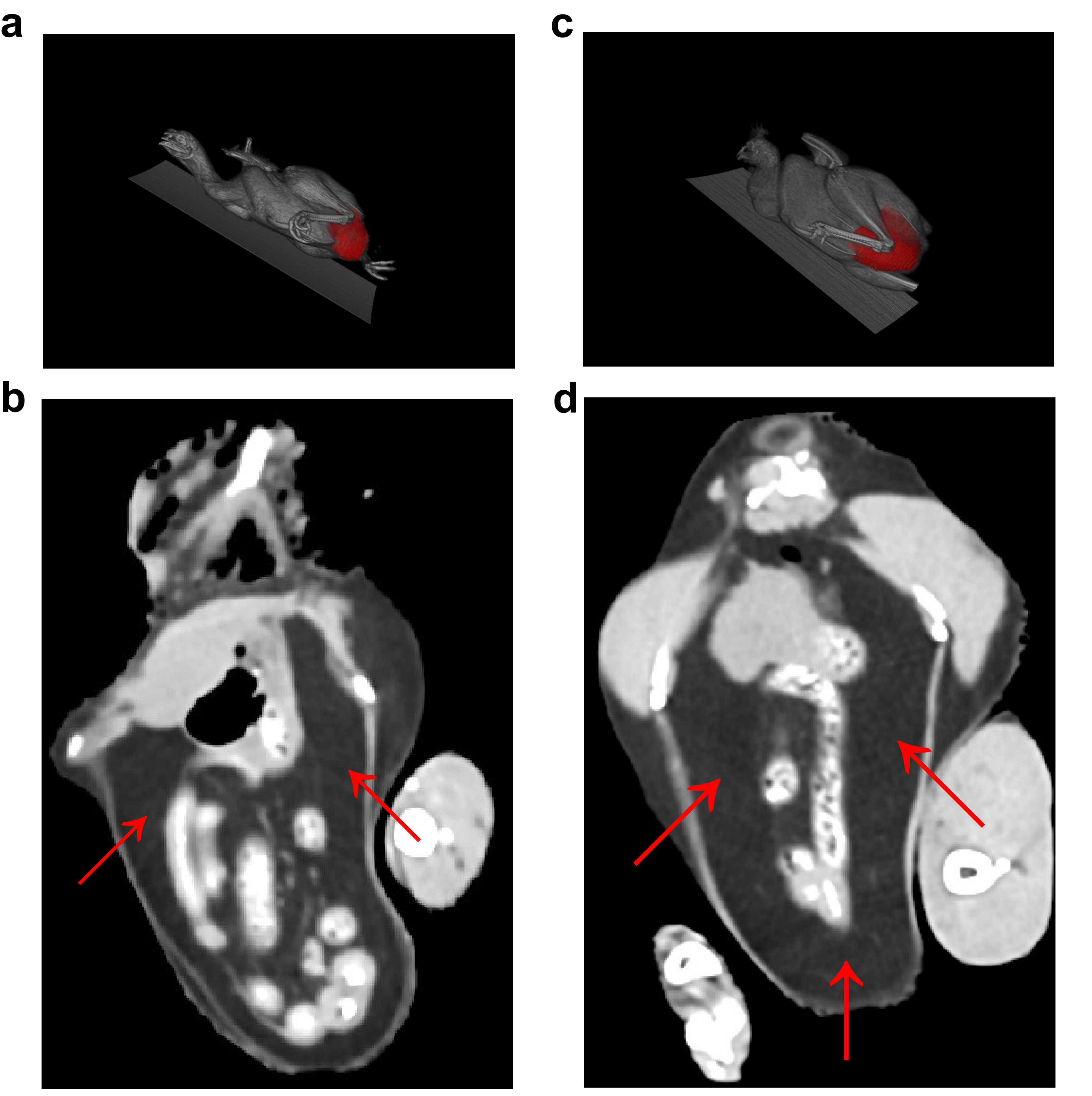

Supplementary Figure 1
Analysis of the fecal suspension donor using CT scanning technique
CT scanning technique was used to analyze the fat deposition-related factors for the donor. A three-dimensional image **a** and a cross section **b** of high body weight and low abdominal fat weight chicken. A three-dimensional image **c** and a cross section **d** of high body weight and high abdominal fat weight chicken. The red area is the abdominal fat in figure **a** and **c**. The gray area indicated by the red arrow is the abdominal fat in figures **b** and **d**.

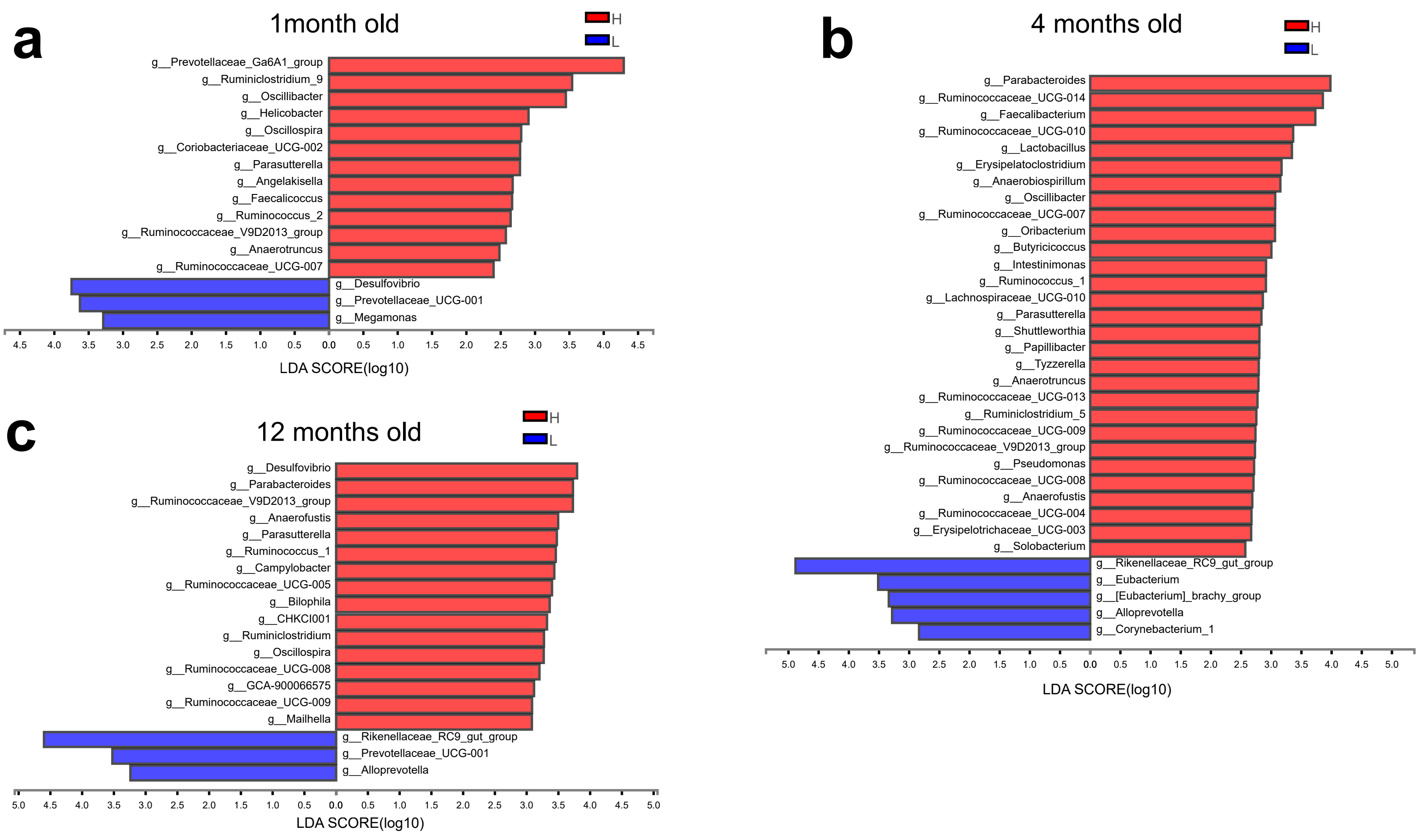

Supplementary Figure 2
Differential microbiota in cecum of high and low abdominal fat deposition chickens
a Analysis of differential microbiota in cecum between high and low abdominal fat deposition chickens at 1 month, **b** at 4 months, and **c** at 12 months. LDA score (log10) > 2.0. H represents high abdominal fat deposition chickens (n = 10), and L represents low abdominal fat deposition chickens (n = 10).

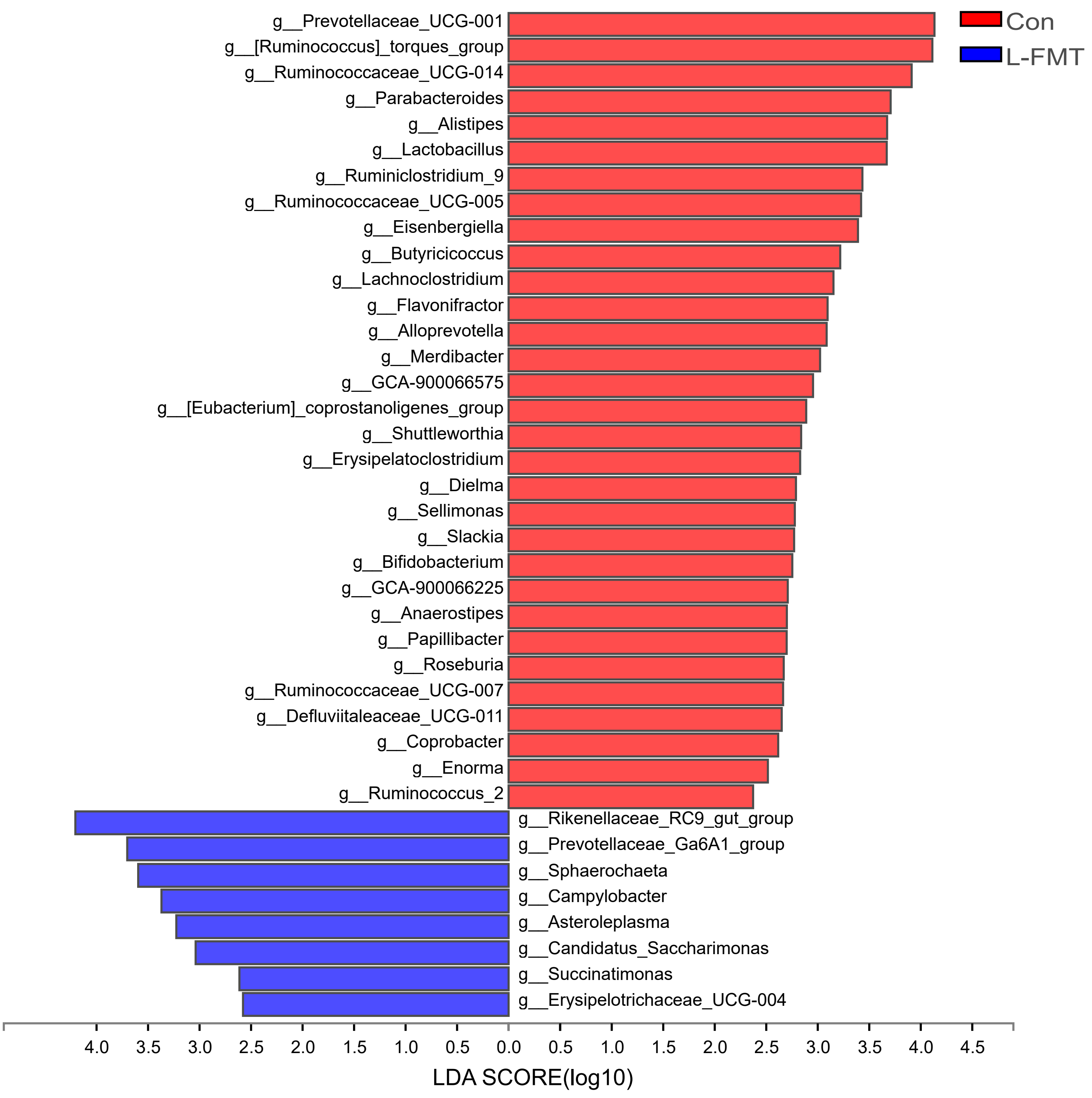

Supplementary Figure 3
Differential microbiota in cecum of the control and L-FMT groups
Analysis of differential microbiota in cecum between the control and L-FMT groups. LDA score (log10) > 2.0. Con represents the control group (n = 14), and L-FMT represents the fecal microbiota transplantation group from the low abdominal fat deposition chicken (n = 14).
